# The G-Protein Coupled Estrogen Receptor (GPER/GPR30) is a Gonadotropin Receptor Dependent Positive Prognosticator in Ovarian Carcinoma Patients

**DOI:** 10.1371/journal.pone.0071791

**Published:** 2013-08-09

**Authors:** Sabine Heublein, Doris Mayr, Thomas Vrekoussis, Klaus Friese, Simone S. Hofmann, Udo Jeschke, Miriam Lenhard

**Affiliations:** 1 Department of Obstetrics and Gynaecology, Campus Innenstadt, Ludwig-Maximilians-University of Munich, Munich, Germany; 2 Department of Pathology, Ludwig-Maximilians-University of Munich, Munich, Germany; 3 Department of Obstetrics and Gynaecology, Campus Grosshadern, Ludwig-Maximilians-University of Munich, Munich, Germany; John Hopkins University School of Medicine, United States of America

## Abstract

Follicle stimulating hormone receptor (FSHR) and luteinizing hormone receptor (LHCGR) were demonstrated to impact upon survival of patients suffering from epithelial ovarian cancer (EOC). Though structure wise the G-protein coupled estrogen receptor (GPER/GPR30) is related to FSHR/LHCGR, its prognostic impact in EOC remains controversial. We recently found that FSHR negative patients represent a specific EOC subgroup that may behave differently in respect to both treatment response and prognosis. Hence, the current study aimed to analyze how GPER may interact with the FSHR/LHCGR system in EOC and whether the prognostic significance of GPER in EOC cases (n = 151) may be dependent on the FSHR/LHCGR immunophenotype of the tumor. Ovarian cancer cell lines were used to study how FSH and LH regulate GPER and whether GPER activation differentially affects in vitro cell proliferation in presence/absence of activated FSHR/LHCGR. In EOC tissue, GPER correlated with FSHR/LHCGR and was related to prolonged overall survival only in FSHR/LHCGR negative patients. Although GPER was found to be specifically induced by LH/FSH, GPER agonists (4-Hydroxy-Tamoxifen, G1) reduced EOC cell proliferation only in case of LH/FSH unstimulated pathways. To the same direction, only patients characterized as LHCGR/FSHR negative seem to gain from GPER in terms of survival. Our combined tissue and in vitro results support thus the hypothesis that GPER activation could be of therapeutic benefit in LHCGR/FSHR negative EOC patients. Further studies are needed to evaluate the impact of GPER activation on a clinical scheme.

## Introduction

Targeting the estrogen receptor (ER) system is a widely accepted strategy in a couple of gynecological malignancies like breast or endometrial cancer. In contrast, in epithelial ovarian cancer the clinical impact of blocking ERs or interacting with estrogen (E2) synthesis is still controversial [1]–[3]. As therapies modulating the classical (DNA-binding) estrogen receptors are of minor clinical importance in EOC, research on alternative receptor systems like the G-protein coupled receptors (GPCRs) is rather up-rising.

GPCRs are membrane receptors that via heterotrimeric G-proteins regulate a wide range of downstream effects including kinase activation and calcium release [4]–[6]. Follicle stimulating hormone and luteinizing hormone receptors (FSHR, LHCGR) are GPCRs that, being predominantly expressed in ovarian granulosa and theca cells, play fundamental roles in reproductive physiology. In response to activation by their respective gonadotropins (FSH, LH, hCG) these gonadotropin receptors (GnRs) regulate follicle recruitment, maturation and growth. Since GnRs have been implicated to also influence proliferation and survival of ovarian cancer cells [7]–[9], they have emerged to be promising targets in ovarian cancer treatment [10]. A former study of our group revealed that EOC patients -positive for either FSHR or LHCGR- show opposing outcomes in respect to their overall survival [11]. FSHR-expressing EOC patients have been related to worse prognosis compared to FSHR non-expressing EOC cases, while LHCGR was found to be a positive prognosticator for EOC survival [11]. In addition since FSHR positivity has recently been hypothesized to determine the prognostic significance of Her2 in EOC patients [12], FSHR expression may be of significance in EOC pathophysiology.

Gonadotropin releasing hormone, being the physiological trigger for LH/FSH secretion, has been highlighted to be down-regulated by estrogen on mRNA level [13]. Since we recently discovered that gonadotropins might regulate the G protein-coupled estrogen receptor (GPER/GPR30) [14] a complex interaction of gonadotropin and estrogen mediated regulation is hypothesized. GPER, formerly known as GPR30, is a GPCR that recognizes estrogen as a ligand and mediates rapid estrogen signaling. So far a wide range of human tissues both of healthy and of neoplastic origin was found to be positive for GPER [15]–[18]. We previously reported GPER to be differentially expressed in healthy ovaries as well as in benign ovarian diseases [18]. However, the prognostic impact of GPER in ovarian carcinoma patients remains at least controversial [19], [20].

Postmenopausal women are characterized by high serum concentrations of LH and FSH both signaling via their corresponding receptor. As the latter were shown to be highly predictive in EOC prognosis, possible correlations among GPER and FSHR/LHCGR were assessed. Being aware of the fact that GPER turned out to might be regulated by LH and at least to some extend by FSH in a primary human granulosa cell model [14], a crosstalk of the GnR system and GPER in EOC was hypothesized. Hence in the current study GPER immunoreactivity was assessed in EOC patients and analyzed in respect to clinicopathological variables, the patient’s GnR immunophenotype and prognosis. To further elucidate a possible crosstalk of the GPER and GnR system we studied the prognostic role of GPER in EOC patients that had been stratified according to their GnR immunophenotype and investigated gonadotropin mediated regulation of GPER in an ovarian cancer cell model.

## Patients and Methods

### Patients

Formalin-fixed paraffin-embedded tissue sections of 151 patients (Table 1) who had undergone surgery for EOC from 1990 to 2002 in our department were included in this study. Histological characterization (serous (n = 106), mucinous (n = 12), endometriod (n = 21) or clear cell (n = 12)) and histological tumor grade according to the WHO criteria were performed by a gynecological pathologist (D.M.). Data regarding clinical stage and survival were retrieved from patients’ charts and from the Munich Cancer Registry, respectively. Most patients (70.9%) presented with advanced stage disease (FIGO III and FIGO IV), 5.9% were staged as FIGO II and 23.2% had early disease (FIGO I). All patients that were staged as FIGO II–IV received carboplatin and paclitaxel as adjuvant chemotherapy. EOC was graded as WHO grade 3 (G3) in 36.0% of cases, while 35.4% were classified as G2 and the remaining 28.6% as G1. Mean overall survival was 7.33±0.6 years; 100 deaths were documented.

**Table 1:** Clinicopathological features.

Clinicopathological features		n	(%)
***Grade***			
	G1	42	(28.6)
	G2	52	(35.4)
	G3	53	(36.0)
***Staging***			
	FIGO I	35	(23.2)
	FIGO II	9	(5.9)
	FIGO III	104	(68.9)
	FIGO IV	3	(2.0)
***Histology***			
	serous	106	(70.3)
	clear cell	12	(7.9)
	endometrioid	21	(13.9)
	mucinous	12	(7.9)
***Age (median) [years]***		58.76	
***Deaths***		100	

### Ethics Statement

All samples were processed anonymously; the study was approved by the Ethics Committee of the Ludwig-Maximilians-University of Munich (approval number: 227-09) and was conducted according to the principles expressed in the Declaration of Helsinki (1975).

### Cell Culture Conditions

The OVCAR-3, SKOV-3 and Caov-3 cell lines were obtained from the American Tissue Culture Collection (ATCC, Wesel, Germany); the Ishikawa cell line was bought from the European Collection of Cell Cultures (ECACC, Salisbury, UK). All cell lines were cultured in Dulbecco’s Modified Eagles Medium (DMEM) containing 10% fetal bovine serum (FBS) without antibiotics/antimycotics in a humidified atmosphere (37°C, 5% CO_2_).

### Immunochemistry

Formalin-fixed paraffin-embedded (FFPE) ovarian cancer tissue sections were stained for GPER as previously described [18]. Sections incubated with rabbit IgG (supersensitive rabbit negative control, BioGenex, Fremont, USA), instead of the primary antibody, served as negative controls while breast cancer tissue sections were used as positive controls as previously described [21]. The signal was quantified by using a semi quantitative method (IR-score) [22] by two blinded examiners. In 14 cases (9.3%), the evaluation of the two observers differed. These cases were re-evaluated by consensus by both observers. After the re-evaluation, both observers came to the same result. The concordance before the re-evaluation was 90.7%.The IR-score is the product of staining intensity (1 = low, 2 = moderate, 3 = strong) multiplied by the percentage of stained cells (0 = no, 1 = less than 10%, 2 = 10%–50%, 3 = 51%–80%, 4 = 81%–100% stained cells). Immunohistochemical data upon FSHR, LHCGR and ERs regarding this panel were retrieved from the laboratory archive as they had been previously published [11], [23]. Median GPER expression (IRS = 8) was set to determine low (IRS≤8) vs. high (IRS>8) GPER expression. To analyze the influence of GPER according to GnR positivity a cut off of IRS = 3 was set to divide the panel into FSHR, LHCGR positive (IRS>3) vs. negative (IRS≤3) [12].

Caov-3, SKOV-3 and OVCAR-3 cells were seeded on glass slides, fixed in acetone for 5 minutes, washed in PBS and blocked using 1.5% goat serum. Rabbit anti-GPER antibody (Lifespan Biosciences, Seattle, WA) was diluted 1∶300 in antibody diluent (Dako, Hamburg, Germany) and incubated overnight at 4°C. The following day samples were processed using the anti-rabbit Vectastain elite kit (Vector Laboratories, Burlingame, CA) according to the manufacturer’s protocol. Finally slides were stained by aminoethyl carbazole (Dako), counter-stained using Mayers acidic hematoxyline and mounted in Aquatex (MerckMillipore, Darmstadt, Germany).

### Gonadotropin Stimulation

On the day before stimulation, cells were seeded into 24 well plates at a density of 7.5×10^4^ cells per well in DMEM/10% FBS. After cells had attached, their medium was changed to FBS-free DMEM and cells were stimulated with rFSH (GonalF, MerckSerono, Darmstadt, Germany) or LH (CellSciences, Canton, MA) at concentrations of 10^2^ U/l and 10^3^ U/l. To mimic pulsatile gonadotropin secretion a second dose at the indicated concentration was added after 24 hours. Following another 24 hour period of incubation cells samples were processed for western blot analysis. In case of RNA experiments, OVCAR-3 and SKOV-3 cells were treated with FSH or LH at concentrations of 10 U/l, 10^2^ U/l and 10^3^ U/l for two hours before RNA isolation was performed.

### siRNA Mediated Silencing of Gonadotropin Receptors

For siRNA knockdown experiments cells were seeded at a density of 1.0×10^5^ cells per well or 4.0×10^5^ cells per well in 24-well (for protein preparation) or 12- well (for preparation of total mRNA) culture dishes, respectively. According to the manufacturer’s protocol (Qiagen, Hilden, Germany) cells were transfected at plating using a total of 75 ng (24-well) or 150 ng (12 well) of the respective siRNAs mix: FSHR: Hs_FSHR_3 (target sequence: AAGAGCCAATATCACAACTAT) & Hs_FSHR_4 (target sequence: TGGCTGCTATATCCACATCTA); LHCGR: Hs_LHCGR_4 (target sequence: AACGTCGGGCTGAACTTTATA) & Hs_LHCGR_5 (target sequence: ACGGCCGGTCTCACTCGACTA). Samples transfected with an equal amount of scrambled siRNA (AllStars negative control) and samples treated with the transfection reagent only (HighPerfect siRNA transfection reagent) were included in each experiment. Both siRNAs and transfection reagent were purchased from Qiagen. To verify a successful knockdown on mRNA level, gonadotropins (LH (10^3^ U/l; SKOV-3), FSH (10^3^ U/l; OVCAR-3)) were added to each well in order to simulate FSH, LH stimulated conditions and samples were simultaneously transfected with the respective reagent-siRNA complexes for six hours before RNA extraction was performed. In order to verify specificity of gonadotropin mediated GPER stimulation on western blot, OVCAR-3 and SKOV-3 cells were transfected as described above. Following a seven hour incubation period the medium was changed to serum free DMEM and new transfection complexes were added for 24 hours. At the same time gonadotropins (LH (10^3^ U/l; SKOV-3), FSH (10^3^ U/l; OVCAR-3)) were added to the respective wells until samples were further processed for western blot analysis. Both a transfection reagent only control and a sample transfected with a scrambled siRNA were included in each experiment.

### Western Blot

Wells were washed twice in ice-cold PBS and lysed in RIPA buffer containing protease inhibitor (both Sigma Aldrich, St. Louis, MO) for 30 min on ice on a shaker. Lysates were spun at 13.000 rpm for 15 min at 4°C and protein concentration of the supernatant was determined by Bradford assay. The Mini-Protean System (Biorad, Hercules, CA) was used for polyacrylamide gel electrophoresis and blotting. PVDF membranes were blocked in 5% marvel in TBS-0.1%Tween20 (TBST) for one hour at room temperature. Rabbit anti-GPER (Lifespan Biosciences; diluted 1∶2000), mouse anti-beta-actin (Sigma-Aldrich; diluted 1∶1000), rabbit anti-FSHR (Abcam, Cambridge, UK; diluted 1∶500) and rabbit anti-LHCGR (Millipore, Billerica, MA; diluted 1∶500) were diluted in 2% marvel TBST and membranes were incubated overnight. Since Ishikawa cells have been previously published to produce GPER [24], [25], they were used as positive controls (data not shown). Membranes were processed using anti-rabbit or anti-mouse Vectastain elite kits (Vector Laboratories, Burlingame, CA) using a chromogenic substrate development protocol according to the manufacturer’s instructions. Each experiment was repeated three times under the same conditions achieving similar results. Blots were quantified by employing the QuantityOne analysis software (Biorad).

### Quantification of *GPER, FSHR* and *LHCGR* Gene Transcription

RNA isolation was performed by using the NucleoSpin® RNA II kit (Machery-Nagel, Düren, Germany). RNA concentrations were adjusted and cDNA synthesis was carried out as described elsewhere [18]. Gene expression per sample was quantified by TaqMan® real time PCR (2 s at 95°C, 40 cycles of 3 s (95°C) plus 30 s (60°C) and finally 30 s at 60°C) employing the following primers (all from Applied Biosystems, Carlsbad, CA): *GPER* (Hs00173506_m1), *ACTB* (Hs99999903_m1), *FSHR* (Hs00174865_m1), *LHCGR* (Hs00174885_m1). Expression of the target gene was determined relative to *ACTB* as a housekeeping gene. Assays were performed three times under the same conditions. Differences in gene expression were calculated using the Rest2009 software [26] and graphics were drawn from Rest2009 output.

### Bromide-deoxy-uridine Cell Proliferation ELISA

OVCAR-3, SKOV-3 and Caov-3 cell proliferation was assessed by quantifying the amount of incorporated Bromide-deoxy-uridine (BrdU) into newly amplified cellular DNA. The assay was performed according to the manufacturer’s (Roche, Mannheim, Germany) recommendations. Cells were seeded at a density of 0.8×10^4^ cells per well into 96-well culture dishes and allowed to attach for four hours. Then cells were stimulated with either 4-Hydroxy-Tamoxifen (OHT), the specific GPER agonist G1 (both Merck, Darmstadt, Germany), Estradiol (Sigma-Aldrich) or the respective carrier solution (ethanol, DMSO) at the indicated concentrations in phenol red-free/serum-free DMEM. Treatment was performed either for 48 (G1, E2) or 24 (OHT) hours, each including a 21-hour period for BrdU labeling. For gonadotropin stimulation rFSH or LH (each at a concentration of 10^2^ U/l) were diluted into the culture media. Experiments were performed three times.

### Statistical Analysis

Data were analyzed employing the SPSS (v21, IBM, Armonk, New York) statistic software. Gamma and Spearman coefficients were employed to correlate data, while the Mann-Whitney U and independent sample Student’s T-test were applied to test for differences between groups. Kaplan-Meier curves were drawn to compare survival times between groups. The chi-square statistic of the log rank (Mantel-Cox) test was employed to test differences in overall survival for significance. Statistical significance for all tests was set as p<0.05 and data was expressed in terms of mean ± standard error (SEM).

## Results

### GPER Correlates with LHCGR and FSHR Expression in EOC

**Figure 1:**
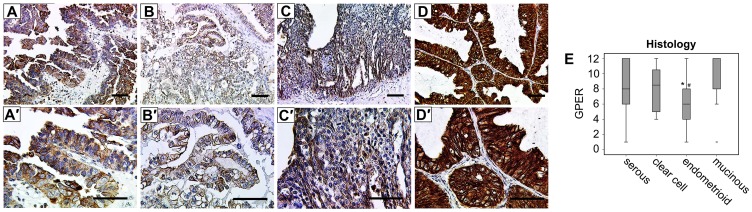
Representative microphotographs of GPER expression in ovarian cancer are presented. GPER showed a membrane as well as a cytoplasmic staining pattern in serous (A, A’), clear cell (B, B’), endometrioid (C, C’) and mucinous (D, D’) ovarian cancer specimens. In endometrioid cancers GPER was significantly lower than in mucinous (p = 0.01, *) or serous (p = 0.03, #) ones. Scale bars equal 100 µm and, box plots present GPER in relation to histological subtype (E). Significant observations derived from relevant Mann-Whitney U-tests.

GPER was strongly expressed with a median immunoreactivity of IRS = 8 (Figure 1). Highest GPER immunoreactivity was observed in mucinous carcinomas (mean IRS = 9.9±1.0, median IRS = 12) while endometrioid tumors (IRS = 6.6±0.7, median IRS = 6) were more weakly stained. Serous (IRS = 8.3±0.3, median IRS = 8) and clear cell (IRS = 8.1±0.9, median IRS = 8.5) EOCs were found to express GPER on a rather moderate level.

GPER was observed to be closely correlated with FSHR (rho = 0.178, p = 0.03) as well as LHCGR (rho = 0.218, p = 0.008) immunoreactivity throughout the panel (Table 2). In serous EOC cases GPER expression was still significantly associated with GnRs (FSHR: rho = 0.242, p = 0.015; LHCGR: rho = 0.255, p = 0.009). Interestingly no relation of GPER and nuclear steroid hormone receptors (ER alpha, ER beta) could be detected (Table 2). GPER expression was significantly elevated in well-differentiated carcinomas as compared to poorly differentiated ones (gamma = −0.325, p<0.001) while a negative correlation of GPER and clinical tumor stage was marginally not significant (gamma = −0.197, p = 0.06).

**Table 2:** Hormone receptor correlations.

			GPER	LHCGR	FSHR	ERα	ERβ
Spear-man'srho	**GPER**	cc	1.000	.178*	.218*	.111	.086
		Sig. (2-tailed)	–	.030	.008	ns	ns

Spearman correlation of receptor expression IR-scores revealed GPER to be positively correlated with expression of FSHR (rho = 0.178, p = 0.030) as well as LHCGR (rho = 0.218, p = 0.008). No relation of GPER and nuclear steroid hormone receptors (ERα, ERβ) could be detected. Stars (*) indicate significant observations; cc = correlation coefficient, ns = not significant.

### GPER is of Prognostic Significance in Gonadotropin Receptor Negative EOC

**Figure 2:**
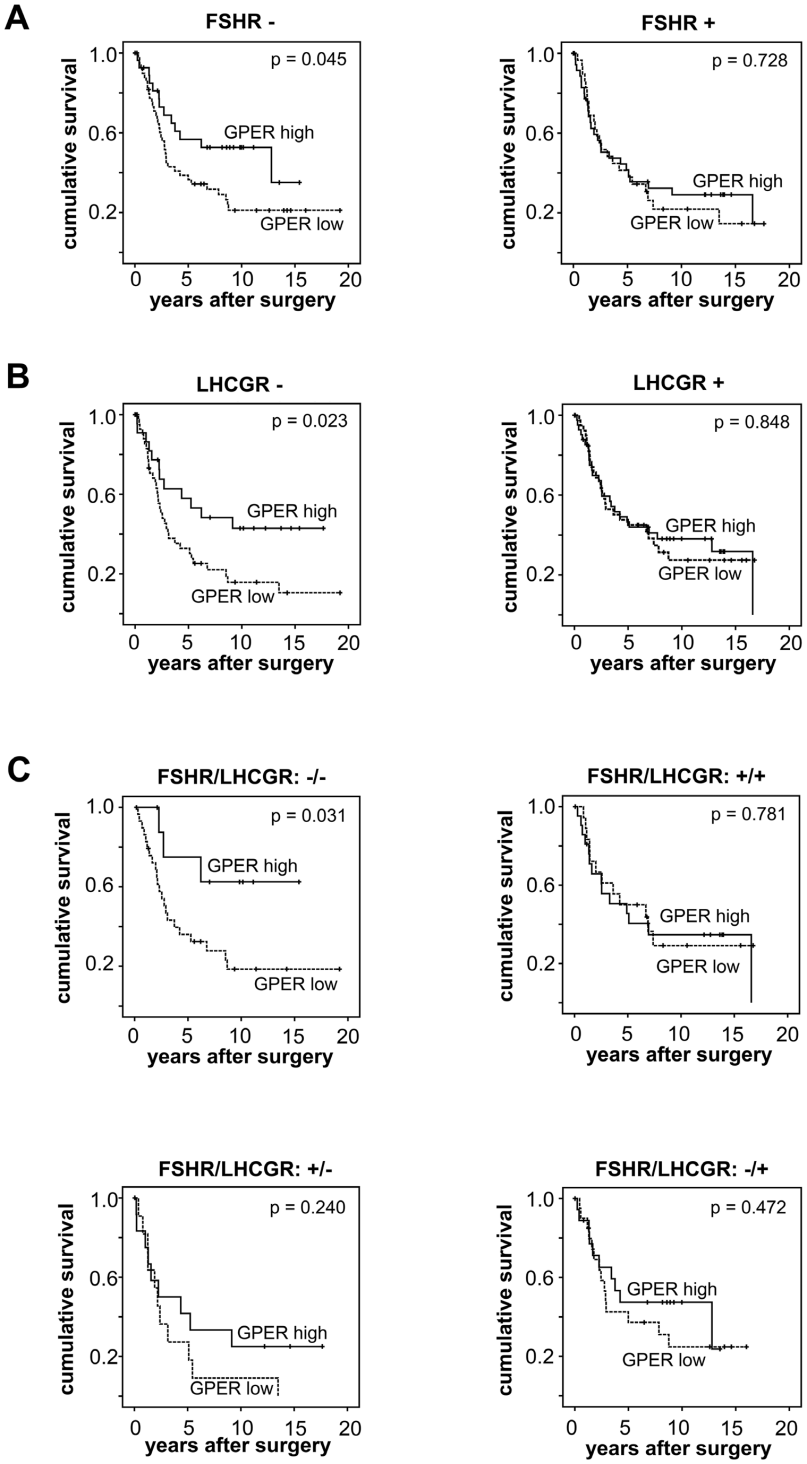
GPER predicts favorable outcome in gonadotropin receptor negative EOC. Prognostic significance of GPER was evaluated in subgroups of patients with or without expression of FSHR or/and LHCGR. Survival of patients whose tumors expressed GPER at high levels (solid lines) was compared to those with low GPER expression (dotted lines) by the log rank test and Kaplan-Meier survival plots were drawn. Remarkably, GPER predicted significantly more favorable outcome in subgroups classified as FSHR negative (A) and LHCGR negative (B). Stratification of EOC patients according the combined LH/FSH status revealed that only in case of a double negative immunophenotype GPER appears as a positive prognosticator (C).

Kaplan-Meier analysis revealed no significant difference in prognosis of EOC patients whose tumors did or did not express GPER (Figure S1). In a previous work we demonstrated LHCGR and FSHR to be independent prognostic markers in EOC and to exert opposing roles on EOC patient survival [11]. This was evident in the present sample as well (data not shown). When FSHR negative cases were evaluated, GPER expression turned out to be related to favorable prognosis (p = 0.045; Figure 2A); the same positive GPER effect was also revealed in LHCGR negative tumors (p = 0.023; Figure 2B). Patients whose tumors presented with a dual negative phenotype (FSH negative/LHCGR negative) - but on the contrary showed GPER positivity - had a significantly prolonged overall survival (p = 0.031; Figure 2C) as compared to the respective GnR positive counterparts. Table S1 shows crosstabulation of GPER, FSHR, LHCGR expression vs. major clinicopathological variables. Crosstabulation of data regarding GPER expression vs. combined FSHR/LHCGR immunophenotype are presented in Table S2.

### GPER is Induced by Gonadotropins in Ovarian Cancer Cells

**Figure 3:**
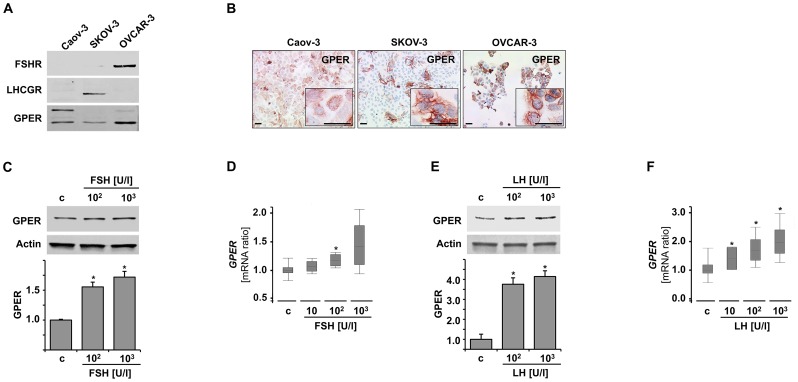
GPER is up-regulated by gonadotropins in ovarian cancer cells. (A) Screening of ovarian cancer cell lines for FSHR and LHCGR. Caov-3 cells expressed gonadotropin receptors at very low to non-detectable levels, while SKOV-3 was LHCGR positive and OVCAR-3 expressed FSHR. GPER was detected in all the three cell lines used in this study (A, B). OVCAR-3 cells were treated with FSH, which enhanced GPER protein expression (C) and gene transcription (D). SKOV-3 cells were treated with LH, which induced both GPER protein (E) and gene transcription (F). c: control, asterisks mark significant (p<0.05) observations as calculated using independent samples Student’s T-test (C, E) and the Rest2009 algorithm for gene transcription ratios (D, F). Scale bars in (B) equal 50 µm.

**Figure 4:**
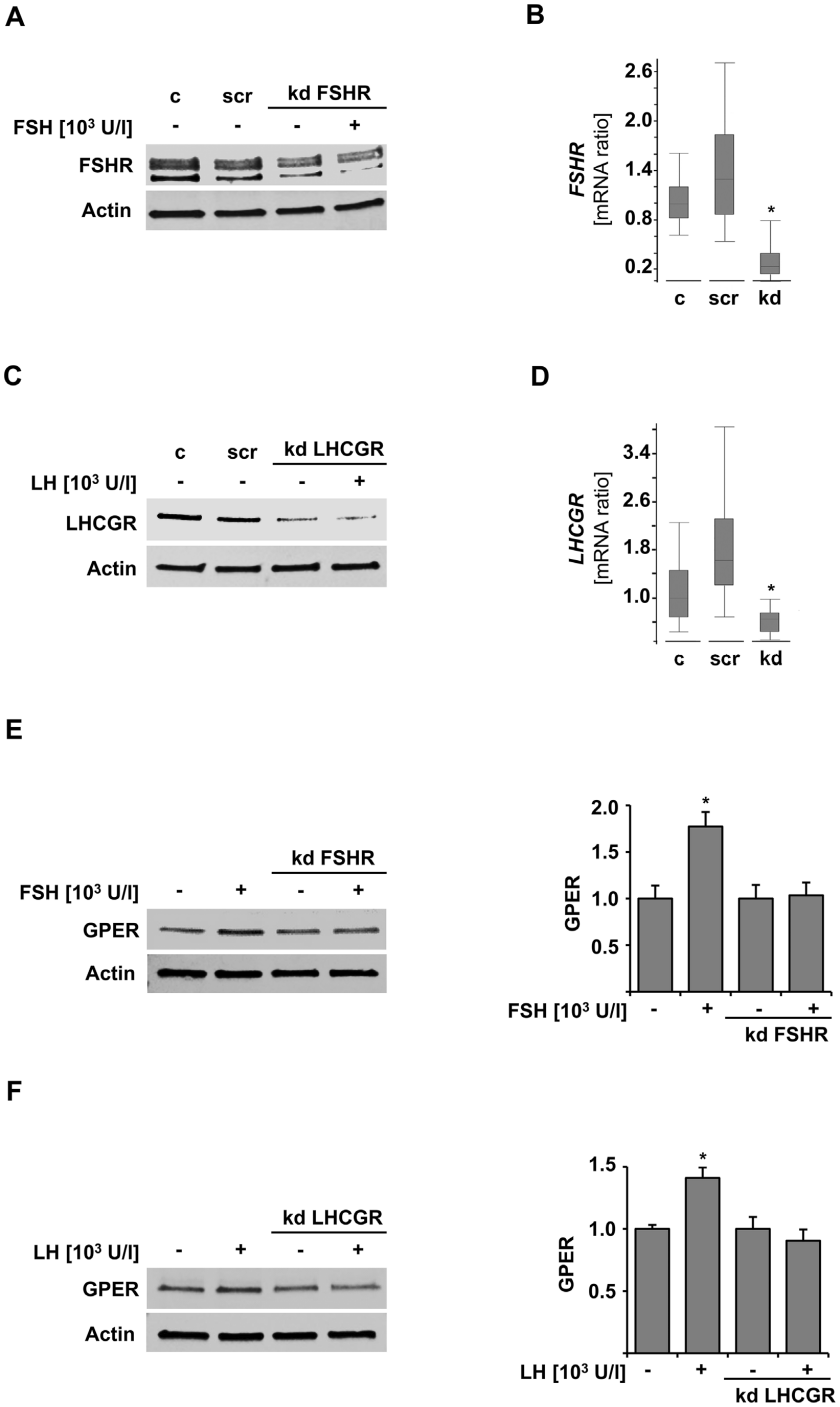
GPER up-regulation is dependent on the respective gonadotropin receptor. siRNA mediated knockdown of FSHR (A, B) and LHCGR (C, D) was performed in order to evaluate whether the up-regulation of GPER is due to an effect specifically attributed to FSH/LH and FSHR/LHCGR. Gonadotropin treatment failed to up-regulate GPER in cells that had undergone FSHR (E) or LHCGR (F) silencing. c: control treated with the transfection reagent only, scr: control treated with an off target, scrambled siRNA. Stars mark significant (p<0.05) observations as calculated using independent samples Student’s T-test (E, F) and the Rest2009 algorithm for gene transcription ratios (B, D).

We performed *in vitro* stimulation assay in two out of three GPER positive ovarian cancer cell lines expressing either FSHR or LHCGR (Figure 3A, B). Cell lines were selected according to their GnR expression as determined by western blot. TaqMan real time PCR analysis was employed to ensure receptor positivity on a gene expression level. Caov-3 cells produced both FSHR and LHCGR protein on a low to virtually undetectable level, while SKOV-3 was found to be positive for LHCGR but not FSHR protein. OVCAR-3 cells presented with the opposite phenotype, being positive for FSHR and negative for LHCGR protein (Figure 3A). *GPER* transcription was significantly elevated (1.2-fold, p<0.001; Figure 3D) in FSHR-expressing OVCAR-3 cells when exposed to FSH. The latter also up-regulated GPER by 1.7-fold (p = 0.016) on a protein level (Figure 3C). Exposure of the LHCGR positive SKOV-3 cell line to LH resulted in a 1.9-fold (p<0.001) induction of *GPER* gene transcription in a concentration dependent manner (Figure 3F). Further, GPER protein was increased up to 4.1-fold (p = 0.001) after 48 hours LH treatment (Figure 3E). In order to prove the selective gonadotropin dependence of GPER stimulation, siRNA-mediated silencing of GnRs was performed (Figure 4A–D). Interestingly, FSH failed to up-regulate GPER in cells that had been silenced for FSHR (Figure 4E) and the same applied for LH when LHCGR was knocked down (Figure 4F).

### GPER Activating Drugs Significantly Reduce Ovarian Cancer Cell Proliferation in a Gonadotropin-Dependent Manner

**Figure 5:**
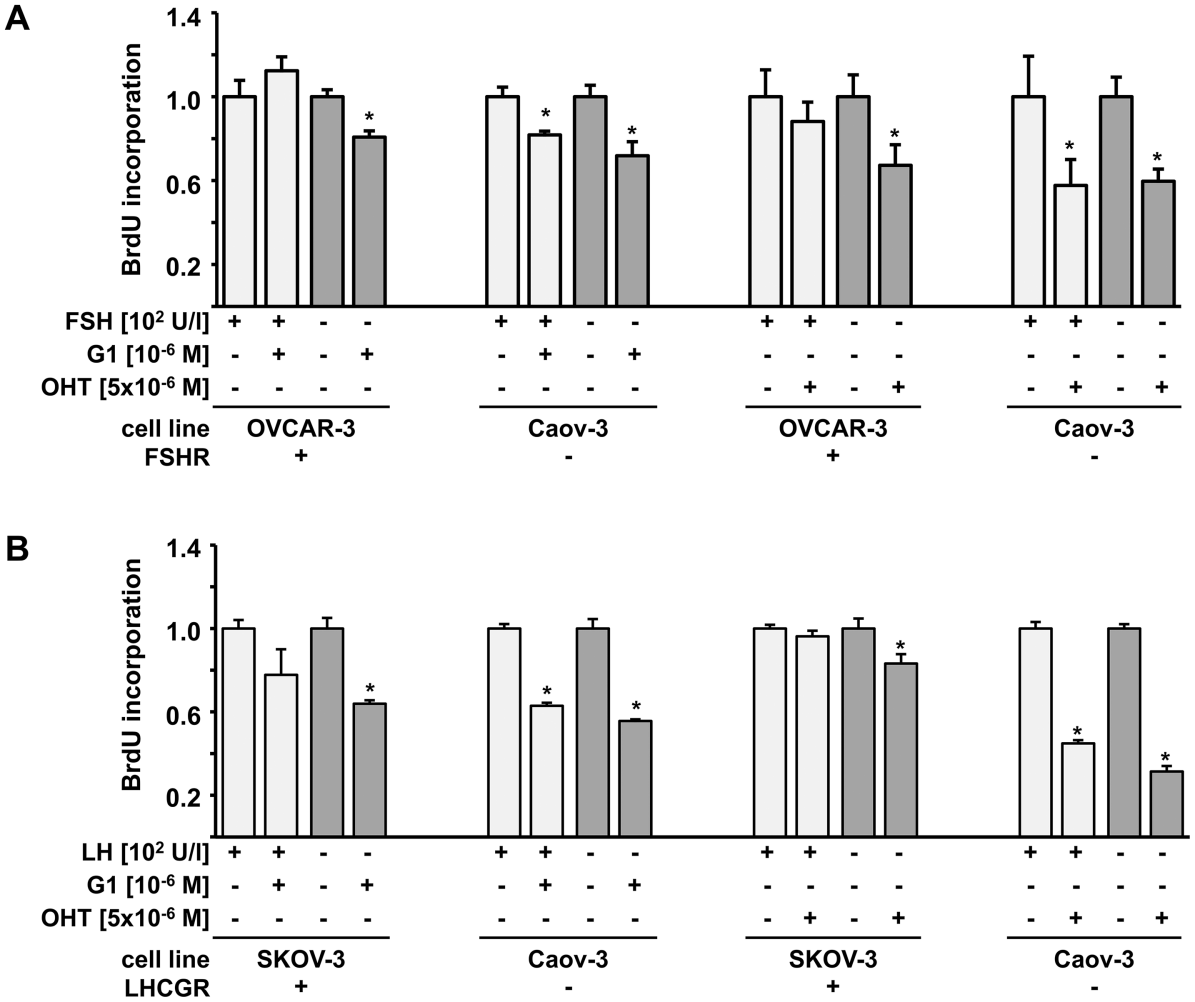
GPER signaling activators inhibit proliferation of ovarian cancer cells lines in an FSH and LH dependent manner. FSHR positive (OVCAR-3) and negative (Caov-3) cells were exposed to GPER agonists (G1; 4-Hydroxy-Tamoxifen (OHT)) in the presence vs. absence of external FSH (A). In the FSH depleted setting G1 and OHT reduced OVCAR-3 cell proliferation by, respectively. Neither G1 nor OHT emerged to significantly affect OVCAR-3 proliferation in the presence of FSH. Caov-3 cells, being negative for GnRs, were sensitive to addition of GPER inducers regardless the presence of FSH, since both G1 and OHT significantly reduced median Caov-3 replication rates. Both G1 and OHT failed to reduce proliferation of LHCGR positive SKOV-3 cells in the presence of LH (B). In LH non stimulated conditions proliferation of SKOV-3 cells was significantly reduced when either G1 or OHT were added to the culture media. Doubling rates of LHCGR negative Caov-3 cells were reduced by G1 as well as by OHT regardless the presence of LH. Independent samples Student’s T-test was employed to test for differences between groups and significant (p<0.05) changes are indicated by stars (*).

FSHR positive OVCAR-3, LHCGR positive SKOV-3 as well as GnR negative Caov-3 cells were exposed to OHT, G1 or estradiol in the presence or absence of FSH or LH. OHT and G1 - being reported as GPER signaling activators [27] - emerged to significantly inhibit proliferation as determined by bromide-deoxy-uridine ELISA. Interestingly, in case of FSHR positive OVCAR-3 cells this inhibitory effect was only present in the absence of external FSH. In the FSH depleted setting both G1 and OHT reduced OVCAR-3 cell proliferation by 19% (G1: p = 0.001) and 33% (OHT: p<0.001), respectively. Neither G1 nor OHT emerged to significantly affect OVCAR-3 proliferation in the presence of FSH. Caov-3 cells, being negative for GnRs, were sensitive to addition of GPER inducers regardless the presence of FSH, since both G1 and OHT significantly reduced Caov-3 replication rates (G1 - FSH stimulated: 18%, p = 0.009; G1 - not FSH stimulated: 28%, p = 0.009; OHT - FSH stimulated: 42%, p = 0.001; OHT - not FSH stimulated: 40%, p<0.001; Figure 5A). LH turned out to produce an effect comparable to that of FSH since both G1 and OHT failed to reduce proliferation of LHCGR positive SKOV-3 cells in the presence of LH. In case of LH unstimulated conditions, proliferation of SKOV-3 cells was significantly reduced when either G1 (36%, p = 0.001) or OHT (17%, p = 0.027) were added to the culture media. However, it needs to be noted that the overall effect of OHT on SKOV-3 cells appeared to be rather low. Doubling rates of LHCGR negative Caov-3 cells were reduced by G1 (LH stimulated: 37%, p<0.001; not LH stimulated: 44%, p<0.001) as well as by OHT (LH stimulated: 55%, p<0.001; not LH stimulated: 69%, p<0.001) regardless of the presence of LH (Figure 5B). Since estradiol is assumed to be the biological agonist of GPER, its effects on ovarian cancer cell proliferation in dependence of gonadotropins were examined within this study. Yet we failed to demonstrate a significant effect of estradiol on ovarian cancer cell proliferation (Figure S2).

## Discussion

Numerous studies report LHCGR and FSHR expression in EOC, though the percentage of receptor positive cases is highly variable [11], [28], [29]. This may at least partly be attributed to the different detection methods used and to the different scoring systems employed. On the other hand, there are only few studies examining GPER in EOC tissue [19], [20], [30]. Despite the different detection strategies applied, they report GPER to be present in at least half of EOC cases. Additionally, Kolkova et al [19] published GPER mRNA to be expressed in a range of ovarian carcinoma cell lines. The current study, using Ishikawa cells as positive controls [24] (data not shown), also revealed GPER to be present in ovarian carcinoma cells on both mRNA and protein level. OVCAR-3 [31] and SKOV-3 [32] cells have already been shown to express FSHR or LHCGR, respectively, while Caov-3 cells produced FSHR on a much lower level [32]. Interestingly though, classical ERs are reported to be rarely found in mucinous EOC [33] three studies have reported GPER positivity in more than half of all EOC samples [19], [20], [30], hence further strengthening a unique role of GPER within the ER family.

The current work is the first to correlate GnR and GPER expression in ovarian cancer. Strikingly, both a positive statistical relation of GPER - GnR in EOC tissue samples and a specific in vitro induction of GPER by gonadotropins was observed. There are several lines of evidence that GPER may interact with the GnR system. Firstly, GPER and GnRs share some sequence features and are members of the same protein superfamily. Secondly, in a recent study on healthy ovaries of pre-menopausal women we identified GPER to be preferentially expressed in theca and granulosa cells [18] commonly regarded as the most prominent target of gonadotropins in premenopausal women. Previously, we found that gonadotropins might be capable of inducing GPER in a model of cultured human granulosa cells [14]. Now, the current analysis detected a gonadotropin-induced GPER up-regulation in ovarian carcinoma cell lines and demonstrated that this up-regulation is dependent on the presence of the respective GnR.

Additionally, we have demonstrated that GPER activation -by the well-established GPER inducers G1 or OHT [27]- was effective in reducing cell proliferation in case of inactive FSH/FSHR or LH/LHCGR pathways. This inactivation was simulated by culturing the cells in the absence of LH or FSH. Further verification upon effective reduction of cell proliferation became evident from the results produced from the Caov-3 cells, being negative for both GnRs. Since the GPER mediated effect on cell proliferation was abrogated in case of either an active FSH/FSHR and/or LH/LHCGR system, it can be hypothesized that, in EOC, apart from inducing GPER expression, LH/FSH signalling may trigger alternative pathways that neutralize the GPER effect on cell proliferation. In that context gonadotropins may exert opposing effects on ovarian cancer cells aiming to maintain cell proliferation.

The presented findings on the GPER mediated action on cell proliferation are in line with observations made in breast cancer studies, where both G1 and OHT have already been reported to inhibit proliferation of breast cancer cells in a GPER dependent manner [27]. In contrast to these results, a recent report hypothesized that G1 may potentiate cell viability of transiently GPER transfected Caov-3 cells, without though showing data on Caov-3 cells expressing only the endogenous GPER [30]. An additional important difference between our study and the one presented by Fujiwara et al [30] was the method applied for cell proliferation assessment. Herein we used bromide-deoxy-uridine labeling to directly quantify DNA amplification, which is inseparably linked to cell proliferation, rather than employing dye based assays that allow estimating the amount of viable cells rather than of proliferation activity.

In the aim of minimizing a possible confounding effect of nuclear ERs (ERalpha, ERbeta) all proliferation assays were carried out in phenol red free and estrogen free conditions in order to reduce baseline activity of ERs. However, it could be argued that a weakness of our proliferation experiment was the lack of demonstrating an effect of estradiol on cell proliferation. This may be possibly interpreted by the complex crosstalk of multiple pathways being initiated by estradiol, via the different ERs mediating opposing signals regarding cell proliferation [34], [35] as well as via non-genomic actions involving a rapid, membrane -mediated activation of ERK1/2 [36]. Moreover, correlation analysis in the EOC patient cohort studied herein demonstrated a significant positive correlation of GnRs and GPER though no such association was observed in respect to GnRs and ER alpha or ER beta.

Prognostic markers that may assist to establish more individualized therapies for EOC patients are rather scarce. Gonadotropins, being abundant in menopause, are suggested to contribute to EOC tumorigenesis [8], [9]. GnRs have been implicated in directly regulating EOC survival [11] and proliferation of ovarian cancer cell lines [8], [9]. Further evidence from three former studies suggests that GnRs exert opposing roles in ovarian cancer [7], [11], [37] with LHCGR being linked to favorable prognosis and FSHR being associated to shorter overall survival. Despite the initial enthusiasm driven by projects referring to prostate or breast cancer [38], [39], the response rates to therapies targeting the hypothalamus-pituitary-ovary axis in EOC turned out to be rather low [10]. This may be explained by the fact that such approaches may have simultaneously negative results on LH and FSH receptor signalling, thus cancelling a negative and a positive prognosticators’ effect in EOC.

ER blocking strategies - by interfering with aromatase enzyme action or by directly antagonizing ERs - have been previously assessed [2], [40], [41]. Though some studies do report that ER based interventions might have positive effects on EOC patients [1], [40], [42] others failed to show such an effect [2]. Hence despite the fact that blocking the classical ER is a widely accepted strategy in breast cancer patients [43], the prognostic impact of blocking estrogen receptors in EOC is at least controversial. However, therapeutic efficacy was difficult to assess since most participants suffered from refractory or platinum-resistant disease, not to mention that properly powered studies were rather rare [3].

The effect of GPER on patients’ prognosis is even less studied. Though initially Smith et al. published that patients highly expressing GPER have significantly poorer prognosis [20], it is now speculated that GPER may not be related to patients’ overall survival at all [19]. Our results also reveal that GPER was not significantly associated with patients’ prognosis when the study cohort was screened without prior stratification according to GnR positivity.

Recently, FSHR was reported to determine the prognostic significance of Her2 in EOC [12], hence strengthening the hypothesis that FSHR negative patients may represent a specific EOC subgroup that might behave differently in respect to both treatment response and prognosis. Hypothesizing that GnR negative patients could indeed represent a special group, the current study found GPER to correlate with more favorable prognosis only in patients that did not express FSHR/LHCGR. The neutralizing effect of LH/FSH signalling on GPER-mediated impact on survival was supported by our tissue culture data: ovarian cancer cell proliferation was profoundly reduced when GPER was activated in case of inactivated LH/LHCGR or FSH/FSHR pathways. The current results, support GPER activation interventions only in case of LHCGR negative/FSHR negative EOC. Further properly powered studies will clarify the exact impact of such interventions.

### Conclusion

In conclusion, in this work it has been shown that, in EOC, GPER expression can be significantly induced by gonadotropins. GPER was found to correlate with GnRs in tumor samples and more importantly to be a positive prognosticator in EOC patients characterized as LHCGR/FSHR negative. This GPER’s impact on survival, combined with the in vitro data demonstrating that GPER activation reduces ovarian cancer cell proliferation in the absence of LH/FSH signaling in vitro, could formulate the hypothesis that GPER activation by e.g. tamoxifen could be of therapeutic benefit in LHCGR/FSHR negative EOC patients. Further studies are needed to evaluate the impact of GPER activation on a clinical scheme.

## Supporting Information

Figure S1 **Effect of GPER expression on EOC patients’ survival.** Kaplan Meier curve presenting the effect of GPER on EOC patients’ survival is shown. GPER-expressing cases do not differ significantly from the non-expressing ones in terms of overall survival.
(TIF)

Figure S2 **Estradiol does not significantly affect proliferation of ovarian cancer cell lines in the chose setting.** Though both OHT and G1 turned out to slow down cell proliferation in a gonadotropin dependent manner, estradiol (E2) did not reveal a significant effect on cell proliferation in our hands regardless the presence of gonadotropins.
(TIF)

Table S1 **Crosstabulation of data and major clinicopathological variables.** Total numbers and percentages of receptor positive vs. negative cases in each subgroup are shown. Receptor positivity was defined as follows: GPER low - IRS≤8, GPER high - IRS>8; FSHR negative - IRS≤3, FSHR positive - IRS>3; LHCGR negative - IRS≤3, LHCGR positive - IRS>3.
(DOCX)

Table S2 **Crosstabulation of GPER and Gonadotropin receptor positivity.**
(DOCX)
