# Supplementary figures and images for: The G-Protein Coupled Estrogen Receptor (GPER/GPR30) is a Gonadotropin Receptor Dependent Positive Prognosticator in Ovarian Carcinoma Patients
Source: PLoS One. 2013 Aug 9;8(8):e71791. doi: 10.1371/journal.pone.0071791 (PMC3739730; doi:10.1371/journal.pone.0071791)

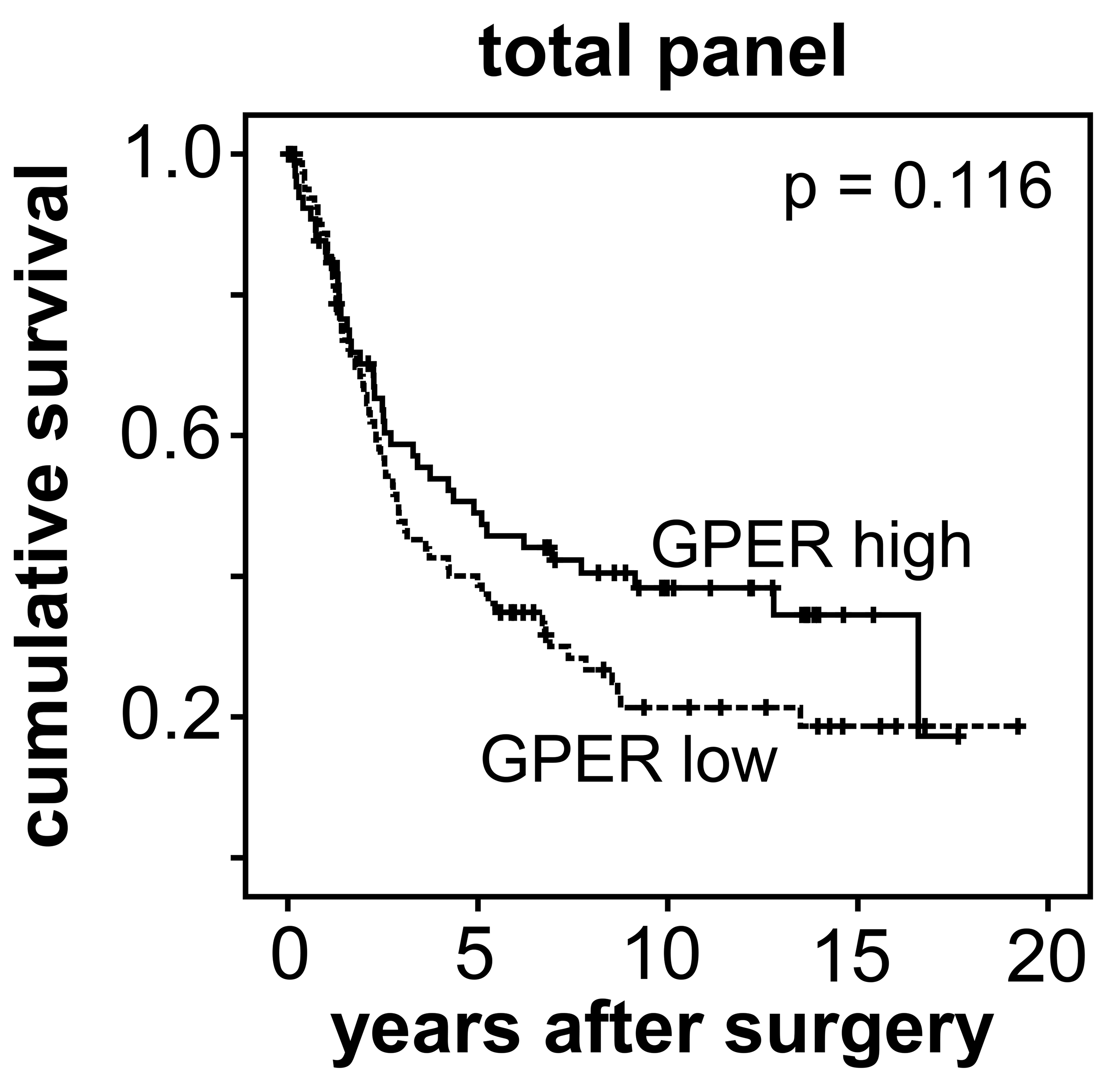

Supplement: Figure S1 — Effect of GPER expression on EOC patients’ survival. Kaplan Meier curve presenting the effect of GPER on EOC patients’ survival is shown. GPER-expressing cases do not differ significantly from the non-expressing ones in terms of overall survival. (TIF) [file pone.0071791.s001.tif]

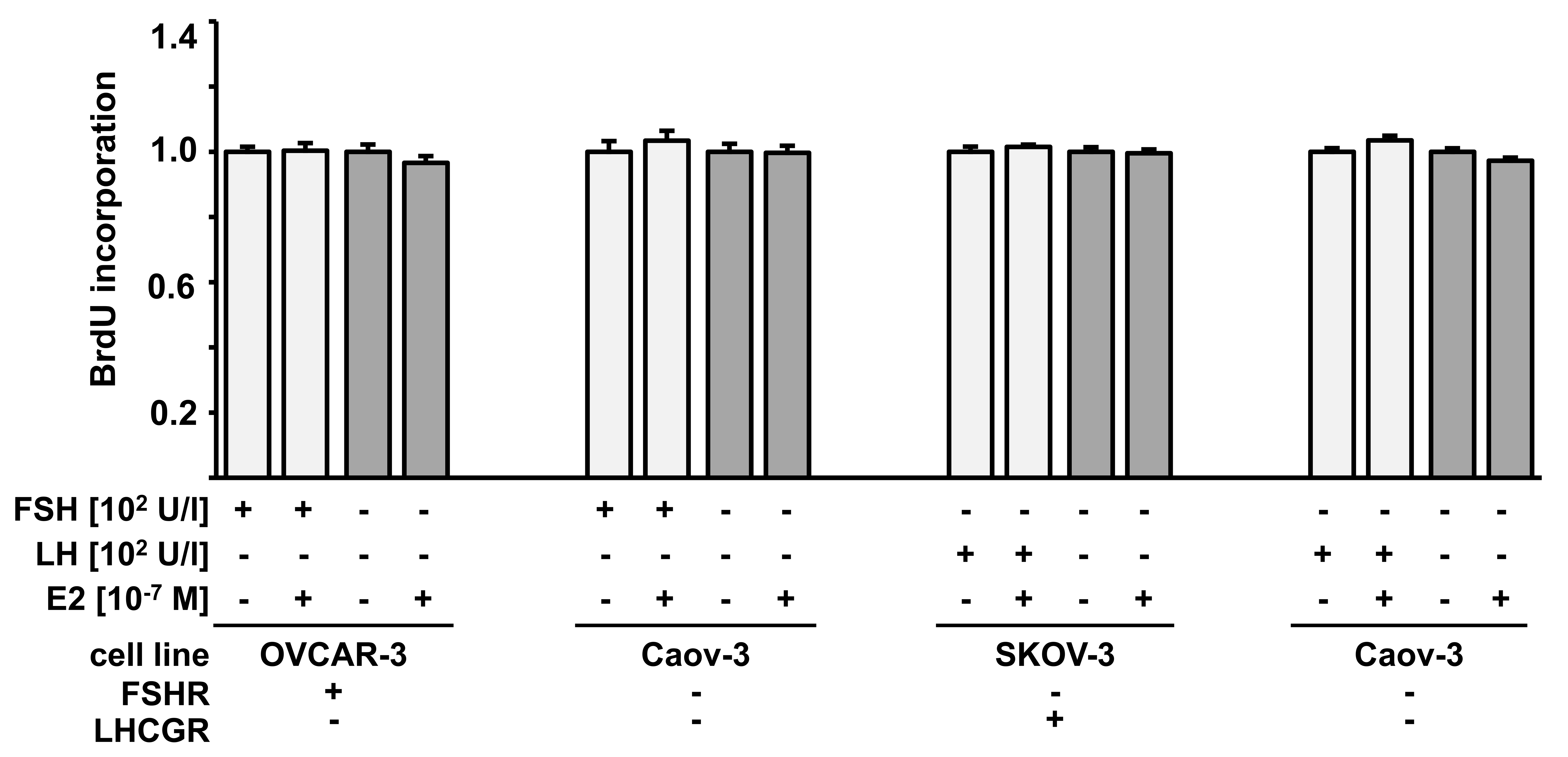

Supplement: Figure S2 — Estradiol does not significantly affect proliferation of ovarian cancer cell lines in the chose setting. Though both OHT and G1 turned out to slow down cell proliferation in a gonadotropin dependent manner, estradiol (E2) did not reveal a significant effect on cell proliferation in our hands regardless the presence of gonadotropins. (TIF) [file pone.0071791.s002.tif]
