# Supplementary material for: The G-Protein Coupled Estrogen Receptor (GPER/GPR30) is a Gonadotropin Receptor Dependent Positive Prognosticator in Ovarian Carcinoma Patients
Source: PLoS One. 2013 Aug 9;8(8):e71791. doi: 10.1371/journal.pone.0071791 (PMC3739730; doi:10.1371/journal.pone.0071791)
Supplement: Table S1 — Crosstabulation of data and major clinicopathological variables. Total numbers and percentages of receptor positive vs. negative cases in each subgroup are shown. Receptor positivity was defined as follows: GPER low - IRS≤8, GPER high - IRS>8; FSHR negative - IRS≤3, FSHR positive - IRS>3; LHCGR negative - IRS≤3, LHCGR positive - IRS>3. (DOCX) [file pone.0071791.s003.docx]

**Supplementary Table 1**

|  |  | **GPER** | | | |  | **FSHR** | | | |  | **LHCGR** | | | |
| --- | --- | --- | --- | --- | --- | --- | --- | --- | --- | --- | --- | --- | --- | --- | --- |
|  |  | low | (%) | high | (%) |  | neg | (%) | pos | (%) |  | neg | (%) | pos | (%) |
| **Histology** |  |  |  |  |  |  |  |  |  |  |  |  |  |  |  |
|  | serous | 57 | (37.7) | 49 | (32.5) |  | 54 | (37.0) | 47 | (32.2) |  | 48 | (32.2) | 56 | (37.6) |
|  | clear cell | 6 | (4.0) | 6 | (4.0) |  | 5 | (3.4) | 7 | (4.8) |  | 6 | (4.0) | 6 | (4.0) |
|  | endometrioid | 16 | (10.6) | 5 | (3.3) |  | 13 | (8.9) | 8 | (5.5) |  | 7 | (4.7) | 14 | (9.4) |
|  | mucinous | 4 | (2.6) | 8 | (5.3) |  | 7 | (4.8) | 5 | (3.4) |  | 6 | (4.0) | 6 | (4.0) |
|  |  |  |  |  |  |  |  |  |  |  |  |  |  |  |  |
| **Grade** |  |  |  |  |  |  |  |  |  |  |  |  |  |  |  |
|  | G1 | 17 | (11.6) | 25 | (17.0) |  | 22 | (15.5) | 17 | (12.0) |  | 20 | (13.8) | 22 | (15.2) |
|  | G2 | 27 | (18.4) | 25 | (17.0) |  | 26 | (18.3) | 25 | (17.6) |  | 16 | (11.0) | 36 | (24.8) |
|  | G3 | 38 | (25.9) | 15 | (10.2) |  | 28 | (19.7) | 24 | (16.9) |  | 30 | (20.7) | 21 | (14.5) |
|  |  |  |  |  |  |  |  |  |  |  |  |  |  |  |  |
| **FIGO Stage** |  |  |  |  |  |  |  |  |  |  |  |  |  |  |  |
|  | FIGO I | 19 | (12.6) | 16 | (10.6) |  | 19 | (13.0) | 15 | (10.3) |  | 13 | (8.7) | 22 | (14.8) |
|  | FIGO II | 4 | (2.6) | 5 | (3.3) |  | 3 | (2.1) | 6 | (4.1) |  | 5 | (3.4) | 4 | (2.7) |
|  | FIGO III | 58 | (38.4) | 46 | (30.5) |  | 55 | (37.7) | 45 | (30.8) |  | 48 | (32.2) | 54 | (36.2) |
|  | FIGO IV | 2 | (1.3) | 1 | (0.7) |  | 2 | (1.4) | 1 | (0.7) |  | 1 | (0.7) | 2 | (1.3) |
|  |  |  |  |  |  |  |  |  |  |  |  |  |  |  |  |
| **Lymph node metastasis** | | | | |  |  |  |  |  |  |  |  |  |  |  |
|  | no | 24 | (25.8) | 18 | (19.4) |  | 25 | (28.1) | 15 | (16.9) |  | 18 | (19.6) | 24 | (26.1) |
|  | yes | 27 | (29.0) | 24 | (25.8) |  | 27 | (30.3) | 22 | (24.7) |  | 21 | (22.8) | 29 | (31.5) |
|  |  |  |  |  |  |  |  |  |  |  |  |  |  |  |  |
| **Age (median=58.76 y)** | |  |  |  |  |  |  |  |  |  |  |  |  |  |  |
|  | ≤ mean age | 38 | (25.3) | 37 | (24.7) |  | 38 | (26.2) | 35 | (24.1) |  | 28 | (18.9) | 46 | (31.1) |
|  | > mean age | 45 | (30.0) | 30 | (20.0) |  | 40 | (27.6) | 32 | (22.1) |  | 39 | (26.4) | 35 | (23.6) |

**Supplementary Table 1: Crosstabulation of data and major clinicopathological variables**

Total numbers and percentages of receptor positive vs. negative cases in each subgroup are shown. Receptor positivity was defined as follows: GPER low - IRS≤8, GPER high - IRS>8; FSHR negative - IRS≤3, FSHR positive - IRS>3; LHCGR negative - IRS≤3, LHCGR positive - IRS>3.
