# Supplementary material for: The G-Protein Coupled Estrogen Receptor (GPER/GPR30) is a Gonadotropin Receptor Dependent Positive Prognosticator in Ovarian Carcinoma Patients
Source: PLoS One. 2013 Aug 9;8(8):e71791. doi: 10.1371/journal.pone.0071791 (PMC3739730; doi:10.1371/journal.pone.0071791)
Supplement: Table S2 — Crosstabulation of GPER and Gonadotropin receptor positivity. (DOCX) [file pone.0071791.s004.docx]

**Supplementary Table 2**

|  | **GPER** | |
| --- | --- | --- |
|  | **Low (%)** | **High (%)** |
| **FSHR** | |  |
| *neg* | 52 (35.6) | 27 (18.5) |
| *pos* | 29 (19.9) | 38 (26.0) |
|  |  |  |
| **LHCGR** | |  |
| *neg* | 43 (28.9) | 24 (16.1) |
| *pos* | 40 (26.8) | 42 (28.2) |
|  |  |  |
| **FSHR/LHCGR** | | |
| *neg/neg* | 31 (21.5) | 9 (6.3) |
| *pos/pos* | 18 (12.5) | 22 (15.3) |
| *pos/neg* | 11 (7.6) | 14 (9.7) |
| *neg/pos* | 21 (14.6) | 18 (12.5) |

**Supplementary Table 2: Crosstabulation of GPER and Gonadotropin receptor positivity**
